# Monitoring and Evaluating Psychosocial Intervention Outcomes in Humanitarian Aid

**DOI:** 10.1371/journal.pone.0157474

**Published:** 2016-06-17

**Authors:** Kaz de Jong, Cono Ariti, Saskia van der Kam, Trudy Mooren, Leslie Shanks, Giovanni Pintaldi, Rolf Kleber

**Affiliations:** 1 Public Health department, Médecins sans Frontières, Amsterdam, The Netherlands; 2 Medical Statistics, London School of Hygiene and Tropical Medicine, London, United Kingdom; 3 Research department, Arq Psychotrauma Expert Group, Diemen, The Netherlands; 4 Department of Clinical and Health Psychology, Utrecht University, Utrecht, The Netherlands; Liverpool School of Tropical Medicine, UNITED KINGDOM

## Abstract

Existing tools for evaluating psychosocial interventions (un-validated self-reporting questionnaires) are not ideal for use in non-Western conflict settings. We implement a generic method of treatment evaluation, using client and counsellor feedback, in 18 projects in non-Western humanitarian settings. We discuss our findings from the perspective of validity and suggestions for future research. A retrospective analysis is executed using data gathered from psychosocial projects. Clients (n = 7,058) complete two (complaints and functioning) rating scales each session and counsellors rate the client’s status at exit. The client-completed pre- and post-intervention rating scales show substantial changes. Counsellor evaluation of the clients’ status shows a similar trend in improvement. All three multivariable models for each separate scale have similar associations between the scales and the investigated variables despite different cultural settings. The validity is good. Limitations are: ratings give only a general impression and clinical risk factors are not measured. Potential ceiling effects may influence change of scales. The intra and inter-rater reliability of the counsellors’ rating is not assessed. The focus on client and counsellor perspectives to evaluate treatment outcome seems a strong alternative for evaluation instruments frequently used in psychosocial programming. The session client rated scales helps client and counsellor to set mutual treatment objectives and reduce drop-out risk. Further research should test the scales against a cross-cultural valid gold standard to obtain insight into their clinical relevance.

## Introduction

There is an urgent need for the assessment of the efficacy of psychosocial interventions in non-Western areas of on-going conflict. Are these interventions worth the efforts and do they really have effects on well-being and health? Although reviews show positive outcomes for certain psychosocial interventions [1], they also point to substantial methodological constraints in current research designs [1] [2] [3]. A major dilemma concerns the frequent use of existing Western self-reporting questionnaires to evaluate intervention outcomes in areas of on-going conflict. Several of these instruments suffer from limitations, including their length, lack of cultural validity and reference to Western mental health concepts. Also, their complexity hinders their application and their use by clients with low levels of formal education.

Little is known about alternative methods to evaluate treatment outcomes from both client and therapist perspectives in non-Western humanitarian settings. In the West client and counsellor rating scales appear to be valid and reliable alternatives for treatment evaluation tools such as self-reporting questionnaires [4] [5]. Therefore, we set out to examine client and counsellor feedback tools (rating scales) implemented in 18 psychosocial programmes world-wide using retrospective data analysis.

The aim of the present study is to assess an alternative for Western self-reporting questionnaires as a method to evaluate client treatment progress and programme outcome in non-Western humanitarian settings. We investigate the validity of a generic method of assessment. Our research questions are: do the client and counsellor feedback tools register changes over the course of treatment, what are the differences between the scales and what variables contribute to these differences? We discuss our findings from the perspective of criterion and concurrent validity.

## Method

Data are gathered in 2009 from 18 psychosocial projects in eight countries. The psychosocial projects are integrated in the services of Médecins Sans Frontières (MSF), a medical humanitarian organization operating in humanitarian contexts.

Data are included from six projects in a ‘conflict’ setting (having experienced active intra- or interstate conflict in the previous 12 months): Colombia (three locations), Democratic Republic of Congo (DRC) (two locations) and Iraq (one location). Three projects in a ‘post-conflict’ setting (a history of armed conflict but no active fighting for at least 12 months) are included: Central African Republic and DRC (two locations). Data from seven projects in an ‘unstable’ setting (political turmoil is present but has not reached the stage of armed conflict) are used: India (three locations), Pakistan (two locations) and Russia (two locations). Lastly, two projects in a ‘societal violence’ setting (high levels of violence not linked to intra- or interstate conflict or political turmoil) are included in the assessment: Papua New Guinea.

The monitoring system of an earlier large-scale mental health project [6] was adapted and developed into the current system. A description of the monitoring system and the general outcomes is published in detail elsewhere [7].

### The study population

All adult (≥18 years), newly enrolled clients in 2009, with more than one counselling session, at least one of three outcomes measures (rating scales) recorded and a closed file, are included in the study. Those included in the analysis received individual counselling as part of a routine mental health programme in one of the 18 MSF psychosocial projects.

### The intervention

The objective of the psychosocial programmes is to reduce psychosocial complaints and to improve (related) functioning [8] [9].

Brief counselling is used to normalise psychosocial reactions to war and disaster, to encourage appropriate expression and containment of emotions, and to mobilise personal resources through identifying resilience mechanisms, strengthening coping skills and activating new problem-solving approaches to address for instance practical problems (see also [10] [11]). Counsellors adopt an empathic solution focused approach in sessions with their clients [12] [13]. Counsellors receive regular follow-up training and clinical supervision by a mental health professional [14].

Counsellors do not prescribe medication. Counsellors are trained on the identification of serious mental health problems. Treatment of patients suffering from these conditions (such as psychosis) is beyond the scope of the counselling programmes. In some projects referral to a local psychiatrist or primary health care physician to provide psychiatric medications is possible. A detailed description of general set up of MSF psychosocial projects is given elsewhere [9].

### Instruments

#### Procedure

We used two client rating scales: one for rating the intensity (severity) of the most important psychosocial complaint and one for rating the degree of functioning. Complaint and functioning rating may overlap but do not necessarily. In emergencies for instance the complaints of people may be substantial while their functioning necessary for survival may not be decreased. Both ratings were done at the beginning of each session (real-time monitoring). Ratings reflect the actual situation at that moment. The client is not asked (but neither forbidden) to compare with previous rating (outcome of previous rating is not shown). Had the rating been done at the end of each session it would have functioned as an assessment of the current session.

The counsellor explains to every new client how to rate the complaint and the level of functioning in daily life. It is also explained that the rating is done every session to assess how the client is doing and adjust the intervention when needed.

The use of the instruments and the interpretation of them have been tested in the local context; directions explaining its use is standardised during counsellor training sessions. Most projects (13, approximately 113 counsellors) have implemented the scaling system and fine-tuned the design of their rating system to their populations the year(s) before the research.

#### Complaint rating scale

Clients present various psychosocial complaints such as nervousness or inability to care for their children. Psychosocial complaints as expressed by the client do not necessarily relate to mental health symptoms (such as anxiety) or syndromes (such as depression). The individual’s definition of the problem, symptoms, functioning, and explanatory model of illness is used as reference to register the psychosocial complaint.

**Fig 1:**
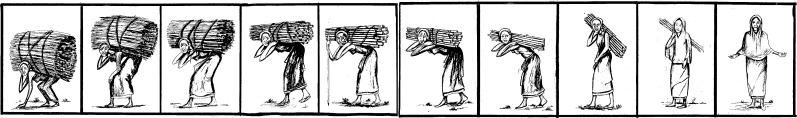
Example of culturally adjusted rating scale.

The clients mark their rating on a scale, which usually is in the form of a line with bars at 1-cm intervals numbered 1–10 from left to right. A mark placed towards the right-hand end of the scale signifies a more positive judgment of the complaint or functioning by the client. In populations with a high level of illiteracy or unfamiliarity with number ratings, the ends of the scale and various intermediate positions are illustrated with local, culturally equivalent symbols (Fig 1).

#### Functioning rating scale

To situate the individual and his problem within his personal context the overall evaluation of functioning was assessed. The Functioning Rating Scale is explained to the client as the degree to which the client can meet the duties of daily life in all its diversity. To rate the functioning the client uses his own frame of reference. Some will use their ability to perform their work, others their capacity to care for their family or to function in social life or combinations.

#### Counsellor rating scale

Status at last visit’ is scored by the counsellor at the end of the treatment. The counsellor scale is rated in relation to the presenting problem for which the client receives treatment. The counsellor can rate the client’s problem as being: completely resolved, improved, unchanged, or deteriorated in severity. The counsellor does not discuss his or her scoring with the client to avoid influencing the score of client score.

### Client and program variables

The following variables related to the patient’s condition are defined: age (per 10 years), gender, precipitating event, counsellor focus, number of sessions, and exit type. The precipitating event associated with the most important psychosocial problem was registered using 11 predefined categories adapted from Hollifield et al. [15].

The counselling focus is classified as clients having practical problems, lack of skills and knowledge necessary for dealing with a difficulty, complaints related to stress (including traumatic stress), being overwhelmed by feelings such as sadness, anger or helplessness, inner problems such as guilt and negative self-esteem and, need for psychiatric support [16].

The following variables, related to project characteristics, are defined: context setting, project size, workload, being professional counsellor, and age of the project.

Workload is defined by the number of clients supported by each counsellor in 2009 (<100, 100–200 or >200 clients per counsellor); all counsellors were full time employed. The size of each project is defined by the number of counsellors in the programme (1–3, 4–6 or ≥7 counsellors).

### Ethical approval

The study is a retrospective analysis of anonymous client information routinely collected as part of mental health services. Patient records/ information were anonymized and de-identified prior data entry and analysis.

It meets the standards set and was approved by the Médecins Sans Frontières Ethics Review Board, an independent international board similar to an ethical board in academic institutions, for retrospective analyses of routinely collected programmatic data.

### Statistical methods

The difference between the first and last client ratings is used to calculate the overall change in the client’s status with respect to their complaints and functioning at exit. For clients who dropped out, the rating of the last one recorded is used.

Mean and standard deviations are used for continuous data. If data are skewed they are presented as median and interquartile range. Binary and categorical data are summarised as number and percentage.

The two client rating scales are analysed using linear regression to estimate crude (unadjusted) associations for analysis of client rating scales, with robust standard errors to account for the heterogeneity induced by clients being clustered within different projects. Multivariable associations of outcomes are assessed using analysis of covariance, again with robust standard errors. Residual analysis is used to check the assumptions of the regression analysis. The eleven categories of main precipitating events were re-categorized for multivariable modelling: psychological violence; physical violence; witnessing violence; sexual violence; displacement related problems; no traumatic experience and ‘other events’. Note that domestic discord or violence which comprises a mixed category of either violence (psychological and/or physical) or discord within the family is classified here as physical violence.

Counsellor assessment at the client’s last visit is analysed using ordered logistic regression, the highest rating being full resolution of a patient’s problem and the lowest a deterioration of the patient’s problem to assess associations. In all cases the models are fitted using robust standard errors. We include exit type in the equations although exit type is not fully independent of the counsellors and client rating as both the client and counsellor can decide on terminating the treatment. We control for this in the multivariable model.

The total number of sessions is entered into the models as a log transformed variable. To control for the specific type of violence from projects in one country (Papua New Guinea: societal violence) a sensitivity analysis is performed on the data set. All data analysis is executed using STATA software, version 12.1.

## Results

### Baseline data

**Fig 2:**
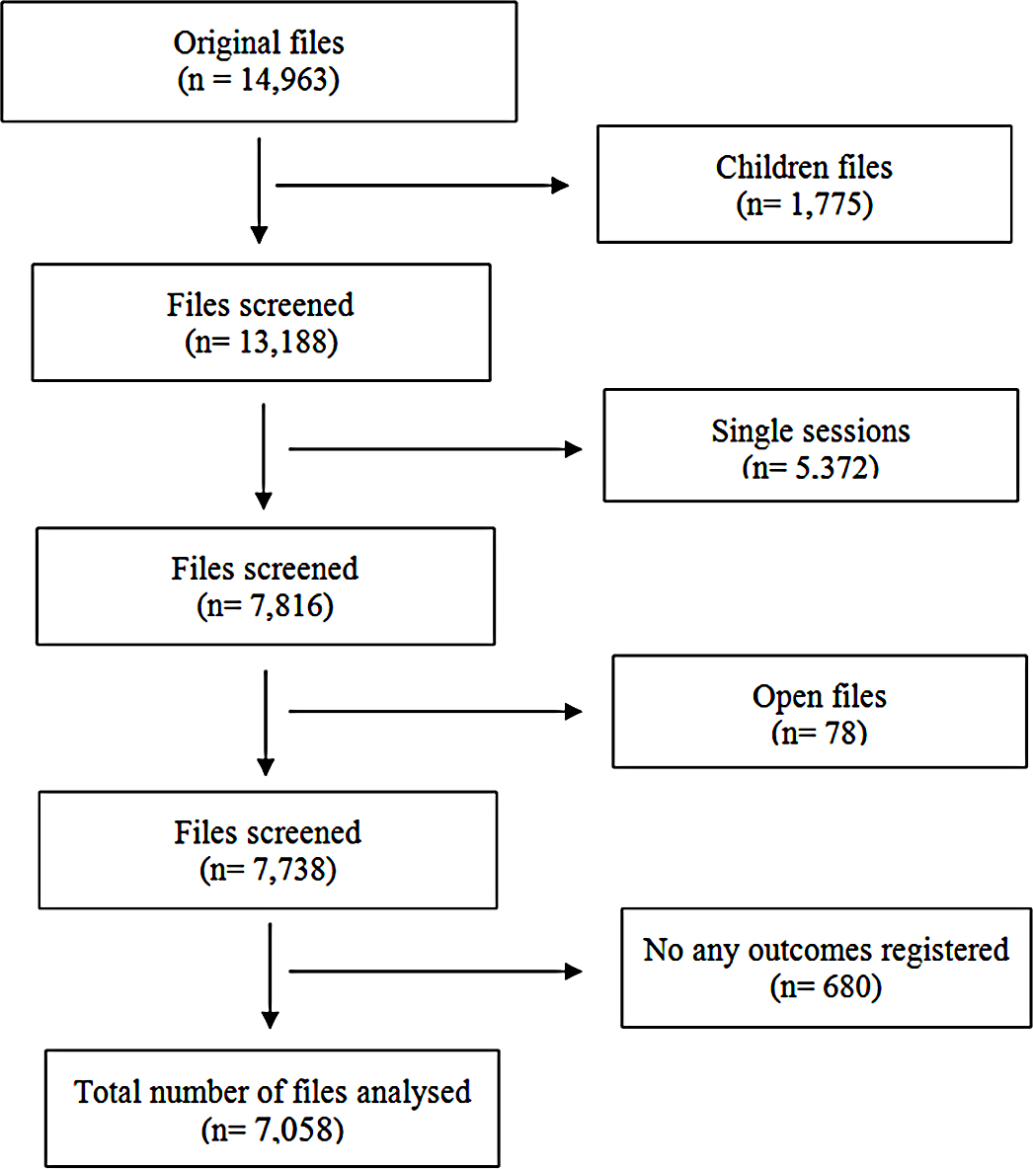
Diagram of files included in final analysis.

Less than half of the clients (7058 of 14,963) are eligible for analysis as shown in Fig 2. Reasons for drop out after one session were mainly related to misunderstanding of the service such as expectations for material support. The majority of the clients included (55.5%, n = 3,915) are located in settings of instability; one-fifth (20.8%, n = 1,470) are living in a conflict zone; those in post-conflict and societal violence settings made up a smaller proportion (11.3%, n = 800 and 12.4%, n = 873, respectively). The study population is predominately female (72.3%, n = 5,101), with an average age of 37.5 years (SD = 12.7).

One-third of the clients presents anxiety-related problems as their main reason to seek counselling (33.3%, n = 2,348). Other frequently mentioned problems are mood-related (16.3%, n = 1,147), family-related (14.6%, n = 1,032) or physical (12.3%, n = 866). Most clients receive treatment from trained, supervised lay counsellors (69.1%, n = 4,878, 11 projects). Counsellors choose as their main counselling focus most often: overwhelming feelings (36.0%, n = 2,543), trauma-related symptoms (24.6%, n = 1,735) and physical complaints (12.3%, n = 866). The median number of sessions is five (inter-quartile range 3–7).

#### Client and counsellor treatment evaluation

Comparing clients’ assessment of their psychosocial complaints and of their function rating at their first visit, a significant difference between the scales is found; the client complaint rating is 0.83 points lower than the functional rating scale (3.34 versus 2.51; 95% CI: 0.52–1.14, p<0.001, n = 7,024).

On average, clients’ rating of their psychosocial complaints post-treatment (complaint scale) show an improvement of 4.8 points compared to that before treatment (7.3 versus 2.5); a similar improvement of 4.3 points is noted for clients’ functional status (7.6 versus 3.3). Comparing pre- and post-treatment ratings, these changes are significant (complaint: 95% CI: 3.8, 5.6; p<0.001; functional status: 95% CI: 3.5, 5.1; p<0.001; both n = 7,007).

Counsellor evaluation of the status of the clients’ psychosocial complaint at the last session (n = 7,039) shows a similar trend in improvement: condition resolved (35.0%, n = 2,468), psychosocial complaint improved (55.9%, n = 3,945). A minority of the client’s conditions are judged as unchanged or deteriorated (7.1% and 1.8% respectively).

### Uni- and multivariable analysis of the change in rating scales separately

Univariable analysis of change within each scale separately shows consistent results for the client (complaint as well as functioning) and counsellor rating scales. The strongest associations of change in all three scales are with the following variables: the type of exit, the total number of sessions and the context setting.

All three multivariable models (see Tables 1 and 2) of the change of each individual scale shows similar significant associations with the independent variables. The strongest association for the client scales (complaint, functioning) are: the rating of the complaint or functional rating at the first visit, the total number of sessions and the exit type. The other independent variables (precipitating event, context setting, counselling focus, size of project) are statistically significant but the estimated parameters are small in absolute magnitude. The variable ‘age’ is statistically significant for the functional scale but not the complaint scale.

**Table 1:** Associations of the individual rating scales and independent variables.

		Linear regression model				Ordinal logistic regression model	
Variable		Estimated Complaint rating change(95% CI)	P-value	Estimated Functional rating change (95% CI)	P-value	Counsellor rating Odds ratio (95% CI)	P-value
Complaint rating at first visit		–0.67 (–0.77, –0.58)	<0.001	N/A**		0.97 (0.83, 1.14)	0.705
Functional rating at first visit		N/A**		–0.67 (–0.75, –0.59)	<0.001	1.14 (1.07, 1.22)	<0.001
Age (per 10 years)		–0.04 (–0.10, 0.01)	0.134	–0.06 (–0.10, –0.01)	0.015	1.00 (0.93, 1.07)	1.000
No. of sessions (log transformed)		1.43 (1.05, 1.80)	<0.001	1.24 (0.87, 1.60)	<0.001	1.94 (1.11, 3.38)	0.020
Exit type			<0.001		<0.001		<0.001
	Drop out *	0.00		0.00		1.00	
	Mutually agreed	1.80 (1.56, 2.03)		1.63 (1.41, 1.85)		13.65 (7.13, 26.12)	
Context setting			0.001		0.001		<0.001
	Conflict	0.71 (0.36, 1.07)		0.48 (0.09, 0.86)		8.31 (2.99, 23.13)	
	Post-conflict	0.03 (–0.25, 0.32)		–0.08 (–0.38, 0.22)		7.05 (2.92, 17.05)	
	Unstable	0.37 (0.08, 0.66)		0.38 (0.10, 0.67)		2.23 (1.15, 4.31)	
	Societal violence*	0.00		0.00		1.00	
Precipitating event			0.010		<0.001		<0.001
	Psychological violence	0.01 (–0.14, 0.16)		0.03 (–0.08, 0.15)		0.84 (0.68, 1.03)	
	Physical violence*	0.00		0.00		1.00	
	Witnessing violence	0.18 (0.04, 0.31)		0.24 (0.13, 0.36)		1.29 (0.99, 1.69)	
	Sexual violence	0.38 (0.07, 0.68)		0.46 (0.14, 0.77)		2.16 (1.61, 2.90)	
	Displacement problems	0.07 (–0.15, 0.29)		0.12 (–0.08, 0.31)		1.04 (0.74, 1.46)	
	Other	0.22 (–0.11, 0.55)		0.27 (–0.06, 0.60)		2.39 (1.08, 5.30)	

Multivariable models: complaint rating change (linear regression model, R^2^ = 0.585, n = 6390), functional rating change (linear regression model, R^2^ = 0.587, n = 6391); Counsellor rating: status at the last visit (ordinal logistic regression model pseudo R^2^ = 0.272, n = 6390).

* = Reference

** = Not included as a model variable

**Table 2:** Associations of the individual rating scales and independent variables.

		Linear regression model				Ordinal logistic regression model	
Variable		Estimated Complaint rating change (95% CI)	P-value	Estimated Functional rating change (95% CI)	P-value	Counsellor rating Odds ratio (95% CI)	P-value
Counselling focus							
	Inner problems	–0.03 (–0.25, 0.18)	<0.001	–0.10 (–0.33, 0.13)	<0.001	1.01 (0.73, 1.39)	<0.001
	Lack of skills & knowledge	0.19 (–0.08, 0.46)		0.14 (–0.05, 0.32)		1.41 (1.03, 1.92)	
	Overwhelming feelings*	0.00		0.00		1.00	
	Practical problems	–0.13 (–0.31, 0.04)		–0.20 (–0.39, –0.01)		0.70 (0.56, 0.88)	
	Complaint related to stress	–0.12 (–0.25, 0.01)		–0.10 (–0.27, 0.07)		0.58 (0.45, 0.75)	
	Psychiatric support treatment	–1.31 (–1.85, –0.76)		–1.34 (–1.90, –0.77)		0.29 (0.12, 0.72)	
Project size							
	1–3 counsellors*	0.00	<0.001	0.00	<0.001	1.00	<0.001
	4–6 counsellors	–0.57 (–1.05, –0.09)		–0.41 (–0.82, 0.00)		0.20 (0.08, 0.49)	
	7+ counsellors	–1.04 (–1.42, –0.66)		–0.70 (–1.01, –0.39)		0.21 (0.10, 0.45)	

Multivariable models: complaint rating change (linear regression model, R^2^ = 0.585, n = 6390), functional rating change (linear regression model, R^2^ = 0.587, n = 6391); Counsellor rating: status at the last visit (ordinal logistic regression model pseudo R^2^ = 0.272, n = 6390).

* = Reference

All the variables in the model, except complaint rating at first visit and client age show statistically significant associations for the counsellor rated scale. The strongest effect size is seen for type of exit and context. The counsellor rating scale has also a strong association, adjusted for the other model variables, with the client’s functional status rating at the first visit.

The strength of associations is largely consistent across the three scales (see for an overview Tables 1 and 2). The multivariable models of the client rating scales are strong in terms of explained variance of the variables (complaint: R^2^ = 0.585, n = 6,390; functional status: R^2^ = 0.587, n = 6,391). The explained variance of the counsellor’s rating multivariable model is acceptable (pseudo R^2^ = 0.272, n = 6,390).

### Differences in change between client self-rating scales

On average clients’ functioning improvement is 0.48 points higher than on the complaint rating scale (crude) (4.75 versus 4.27; 95% CI: 0.16, 0.80; p<0.001, n = 7,004). After adjusting for rating-scale baseline differences, the functional rating scale change is 0.19 points higher (95% CI: –0.27, –0.11; p<0.001, n = 7,004) than the complaint rating scale change.

**Fig 3:**
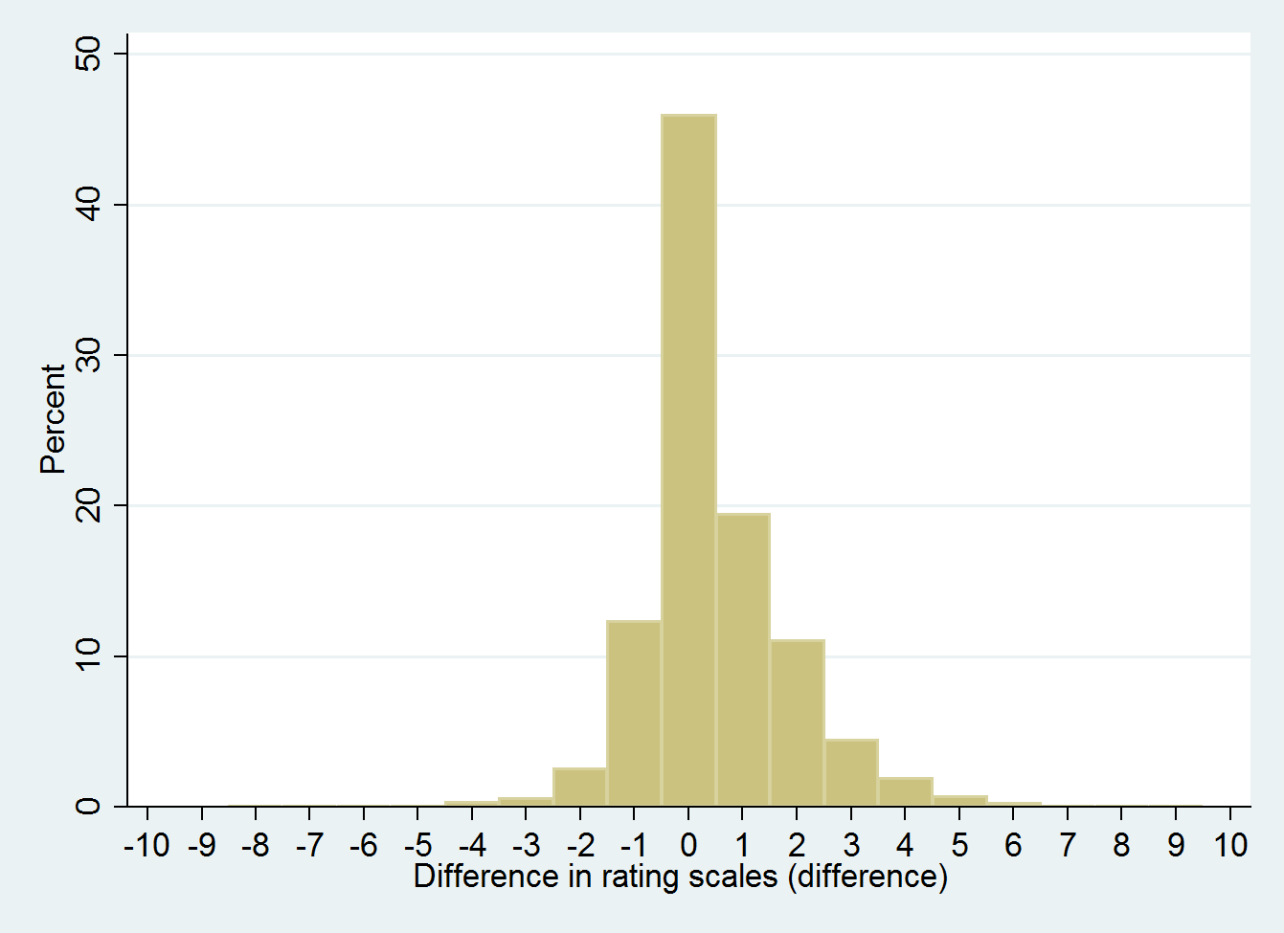
Efficacy in monitoring change of two client rating scales used to assess counselling outcome. Histogram shows difference in the changes in the complaint and functioning rating scales. Rating scale difference is defined as (complaint rating at client’s last visit–complaint rating at the first visit)–(functioning rating at the client’s last visit–functioning rating at the first visit).

Nearly half (46.0%, 3,219, n = 7,004) of the clients has the same change for both the complaint and the functional rating scales (Fig 3). A small proportion (12.3%, 861, n = 7,004) of clients has a functional rating change one point higher than their complaint rating change, and 19.5% (1,363, n = 7,004) has a complaint rating change one point higher than their functional rating change. For most of the clients (77.8%), the change in rating scales differs by no more than one point between the complaints and functioning scale.

Multivariable analysis reveals that the difference in change between the two client scales is related strongly to the different ratings in scales at the first visit. Similar to changes in the separate client scales analysis, the total number of sessions, context setting and the size of the projects are associated (all p<0.001) but their effect on the difference between the scales is small. The multivariable model explains 61.5% of the variability of the differences in the changes of the rating scales.

### Associations between client rating and counsellor assessed client status at last visit

**Fig 4:**
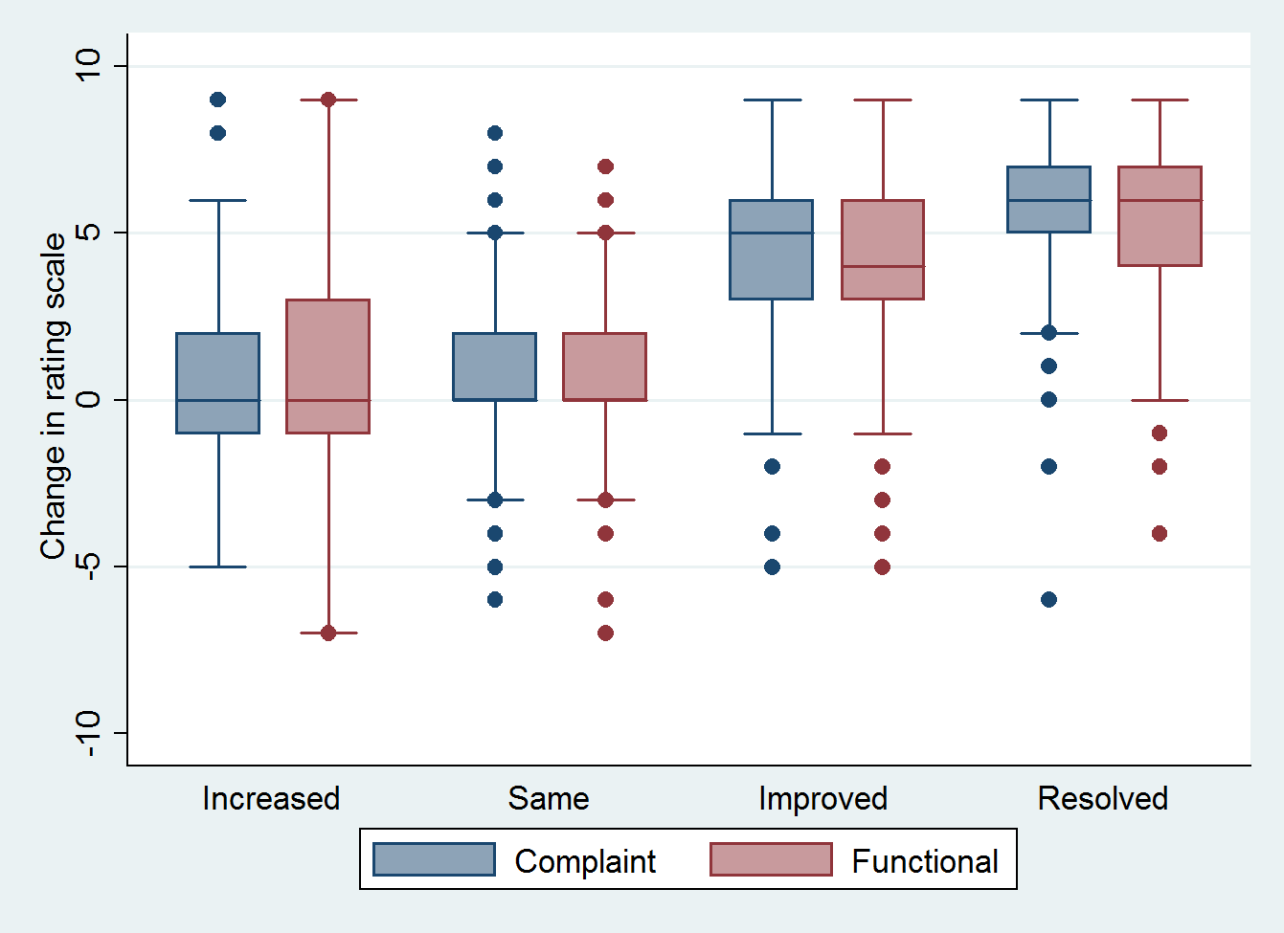
Association of the more favourable counsellor-assessed outcomes with larger improvements in the client rating scales. Box-plot shows the distribution of change in rating scales (complaint, functioning) for each status (increased, same, improved, resolved) at client’s last visit as assessed by the counsellor (n = 6,988).

More favourable counsellor-assessed outcomes are associated with larger improvements in each of the client rating scales (Fig 4).

The association between counsellor-assessed status and the complaint rating improvement of the client shows a similar pattern. Resolved status is associated with a large improvement in client complaint rating (5.10; 95% CI: 4.25–5.92) and improved status links with a moderate client rating improvement (3.04; 95% CI: 2.30–3.71). When the counsellor’s assessed status is deteriorated, the mean deterioration in client complaint rating is small (0.01; 95% CI: –0.93, 0.95).

Post hoc analysis shows a significant difference in the complaint rating scale change between counsellor-assessed status of ‘resolved’ and ‘improved’ (2.05 95% CI: 1.23–2.87, p<0.001), but there is no evidence of a difference between clients complaint rating whose problem remains unchanged and those who are worse off at the end of treatment (p = 0.975).

The client functional rating improvement is 4.56 points when the counsellor assessed the client’s problem as being resolved (95% CI: 3.85–5.27). A counsellor’s assessment of the client’s complaint as ‘improved’ is associated with a client functional rating improvement of 2.82 points (95% CI: 2.12–3.53). Clients assessed by the counsellors as deteriorated show a small deterioration in their functional rating: –0.09 (95% CI: –0.99–0.80).

Post-hoc analysis shows a significant difference between the functional rating change for clients with resolved versus improved condition according to the counsellors assessment (1.74 95% CI: 0.99–2.48, p<0.001). There is no evidence of a difference between patients whose problem remains the same versus those whose problem worsened (p = 0.836).

The sensitivity analysis on the data set from Papua New Guinea (societal violence) reveals a slightly poorer fit in the prediction of the rating scales by the counsellor-assessed status, but the strength of associations remains very strong.

## Discussion

Data from 18 psychosocial programmes in non-Western settings were used to evaluate a generic method of assessment by means of two client and one counsellor treatment progress rating scales. No major differences between the client scales and the counsellor rating scale were found. All scales showed similar degree of change. Also, variables associated with change (within the scales) were comparable. The discussion will focus on the implications of our findings for the construct and convergent validity of the scales and their usefulness for future psychosocial interventions.

### Criterion validity

Various findings point to a good criterion validity. Firstly, treatment interventions are expected to result in changes in the clients’ condition. All scales show a comparable improvement of the client’s condition. The multivariable models applied to all scales show similar associations between client and project characteristics. The total number of sessions (median 5) and the agreement of both the counsellor and client to terminate the support (type of exit) explained most of the change in outcomes. The modest number of sessions as well as the mutual agreement to exit may hint at clients’ suffering from moderate complaints. This result is in line with the target population of most of our psychosocial services. Mutual agreement on exit infers also a good working relationship between client and counsellor which is known in other settings to be an indicator for therapy success [17].

Multivariate analysis showed significant relationship between the scales change and the following variables: precipitating event, the type of context, the focus of the counsellors’ intervention and the size of the project. However, explained variance of the relationships were small. This may be interpreted in that the changes in scales scores are similar regardless of the type of precipitated violent experience, the context of the project (ranging from conflict to post-conflict), type of intervention, or the size of the project. This may mean counselling services can be implemented successfully in very different contexts and situations.

Client rating changes can be interpreted as an indication of client satisfaction. Also, from this perspective the similarity in the changes and the associations of the scores with other variables strengthen criterion validity. Besides, several studies show that client satisfaction is associated with treatment gain [18] [19].

In Western non-conflict settings a dose–effect relationship for the number of treatments has been found generally [20] [21]. The number of sessions necessary to create change in our client population is consistent with the findings of these studies. The median number of sessions in our sample is five. Findings from other studies in Western non-conflict settings indicate that change is likely to occur after a minimum of three and a maximum of 10 sessions [22]. Howard et al. [23] found that 60–65% of people experience significant symptomatic relief within seven visits, figures that increased to 70–75% after 6 months, and 85% at 1 year. The findings clearly indicate that the number of sessions showed an important association with the change in both client and counsellor ratings. This treatment dose–effect relationship supports the criterion validity of the scales.

### Concurrent validity

Two different scales (complaints, functioning) were used to establish the most important drive for clients to visit counselling services. The change in the functional scale was 0.48 higher than the change in the complaint scale (4.3 versus 4.8 points). After adjustment for the significant difference at first visit, the complaint rating was 0.19 points higher which is essentially too small a difference to have programmatic implications.

Interestingly, the significant worse complaint score compared to the functional score at first visit suggests that the alleviation of their complaints may be for clients a more important reason to seek counselling, with their compromised functioning being a weaker drive. Potentially, this finding is important for contexts in which psychosocial and mental health services need to be promoted, as is the case in most parts of the world. To focus service awareness messages (psycho-education) on complaints rather than on the functioning appears to be an useful approach. But when evaluating the benefit of the treatment clients still gave a better final rating to their functioning, though their change in scale was a bit smaller than the change in complaint scale. This may be the result of the problem-solving approach adopted by our counsellors. It certainly shows that for the evaluation of psychosocial and mental health complaints improved functioning from the client’s perspective is an important indicator of change next to complaint improvement. More importantly, in areas of on-going violence, improvement of functioning from the client’s perspective may be even essential for survival of the clients.

We found that clients expressed their distress in terms of either functioning or complaints and there was no major difference between these ways to express. Based on our findings the use of only one client rating scale may be sufficient. However, we suggest for future research to include both the functional and complaint client rating. A potential difference between the two types of client ratings should be investigated further in other (controlled) studies.

To determine concurrent validity asks for comparison with an accepted Gold standard. In the West clinician-rated measurements of improvement are an accepted Gold standard [24]. Using our counsellors rating as standard we found similar changes in the client rating scales. More favourable counsellor-assessed outcomes were associated with larger changes in each of the client rating scales after adjusting for the baseline severity.

Some Western studies [25] have disputed the clinician rated measurement as an accepted standard. They found low correlations between self-ratings and therapist ratings, concluding that clients’ and therapists’ assessment of the quality of the therapeutic alliance may differ considerably. Patients may overrate themselves relative to clinicians [26]. In addition to a higher number of sessions the large congruence in our study may be explained by the continuous monitoring we applied compared to two single pre- and post-measurements. There are some indications that the use of continuous client feedback system compared to treatment as usual improves treatment outcome[27]. Longitudinal rating allows relationships, objectives and expectations to be adjusted over the course of the treatment leading to more congruent assessments. Active engagement of the client in his or her counselling process may also result in greater similarity between counsellor and client outcome ratings.

### Implications

The relevance of this study is the development of an alternative for the Western, disorder-oriented self-reporting questionnaires often used for client treatment evaluation in psychosocial programming in contexts of manmade violence and natural disasters. The tool that we evaluate is useful for both regular, on-going programme monitoring as well as for overall programme efficacy evaluation.

The scaling method registers clients and their problems in their ecological context using their own reference of what is problematic and how it affects what they value as important areas of functioning. Their own local language is used in problem definition and evolution assessment. Diagnostic categories can be developed from this and result in patterns of phenomena observed in general populations [28]. Some (e.g. [29]) see the usage of non-specialist labels as essential for accessible and effective mental health interventions.

The scaling method also allows for a dimensional approach opposed to the binary ‘sick/not sick’ method displayed in Western diagnostic systems (e.g. DSM). As such it may allow for earlier intervention and more precise evaluation over time of the problem than regular diagnostic systems in which first the threshold criteria must be met. We see this scaling method as a potential promising start for the development of a new paradigm of Network Mental Health [30].

Client feedback tools should be simple and brief [31]. Our client rating scales were easy and quick to implement in different countries and cultures. Adaptation of the physical presentation of the scales for each context is simple. Although at the start counsellors had difficulty explaining to clients the difference between complaints and functioning, through training, clinical (group) supervision, and team meetings the best practices are shared and standardized. Involvement of local counsellors in the design ensures the scales’ face validity with the clients. This feature is often missing in longer and more technical measures such as psychological diagnostic and screening questionnaires commonly used in psychosocial programmes.

In the course of our intervention we also observed treatment quality improvements caused by the client evaluation tool. The counsellors appreciated the use of the client rated scales, as it gave them an objective measure of the client’s status at the start of each session, and helped them keep the focus on the client’s main concern. Western research shows that on-going client monitoring is relevant for the immediate quality improvement of the treatment intervention [17]. In line with other research [32] our (non-systematic) field observations confirmed that that the continuous client feedback strengthened the therapeutic alliance between client and counsellor. The therapist’s sensitivity to the client’s subjective world is essential in cross-cultural settings. A recent meta-analysis has identified the therapist’s ability to adapt his approach to the client’s explanatory model of illness as the sole variable that explained superior outcome in culturally adapted psychotherapy [33].

The on-going monitoring helped our counsellors to predict early drop out. Our experiences in the field while working with this monitoring system approach supported the research showing that a client’s subjective experience of meaningful improvement in well-being after the first three sessions of therapy predicts successful treatment outcome [34] [31]. Furthermore, clients worse at the third visit were twice as likely to drop out of treatment [22]. Use of short client feedback instruments developed in Western countries for continuous measurement of the (changing) treatment process has been proven to enhance treatment outcome and reduce drop out [35] [36]. Our counsellors used the feedback of the client scales and discuss changes (or the lack thereof) with their clients. Challenging clients with no improvement of their condition were presented at the clinical supervision sessions to seek consultation from colleagues. Supervisors used the changes of the client scales to monitor quality of the counsellors. Counsellors having unusual changes (very high or very low) were invited to look into this phenomenon.

The positive findings of our study, implemented in 18 projects, may add a new important dimension to psychosocial programme evaluation in emergencies. Though our proposed method of monitoring does not inform on specific changes in pathology it adds the dimension on what may be equally important: whether it is perceived as useful for our beneficiaries.

### Limitations of the study

Our analysis of project monitoring data has also limitations. We have to be careful with the interpretation of the positive treatment outcomes. The outcomes refer only to the client populations under treatment; a comparison population was not part of the analysis. It is outside the scope of this retrospective study that used regular programme data to compare against a reference population of waiting-list clients. Also, we have left out about half of the client population who has had only one session (for an overall review see Shanks et al. [7][37]). The reasons for exclusion one session files are appropriate because most clients have a different expectation of the service (economic support). Also, one measurement does not allow for an evaluation of the development on the scales. However, the exclusion may have influenced the analysis of our ratings and comparison between the scales at first visit. Furthermore, in future studies we suggest to include the variable ‘treatment time’ as the length of treatment may be variable in the healing process.

As mentioned above the use of brief self-rating scales has shortcomings [38]. In the analysis of the treatment changes, a potential ceiling effect is present for clients with a high score on the pre-treatment scales. Furthermore, the assessment depends on both the counsellor’s correctness regarding the accurate evaluation of the client’s psychosocial complaint at the end and the accuracy of the client’s self-rating. Potentially, this accuracy is influenced by the usage of a ten-point scale. Especially, in populations unfamiliar with number rating this may be problematic. However, we think we have addressed this problem by using contextualised pictures in which client are able to recognize themselves.

A limitation in determining concurrent validity is that we did not have a validated scale to compare with our rating tools. Hence, it is difficult to relate our findings to outcome literature from the West because we do not know how the severity of our clients’ psychosocial problems relates to those clients in the literature based on Western contexts.

Furthermore, our sample size is large and significant differences may be found in cases where the actual change is relatively small, producing results that are statistically significant but not clinically meaningful. Both hinder the specific and detailed interpretation of what is a meaningful change of the scales. We have the following suggestions to improve our understanding of the changes in the scales and concurrent validity.

Firstly, the most common method of comparing results is the application of a gold-standard instrument. Unfortunately, in most non-Western settings cross-cultural validated gold standards do not exist; at best they are locally calibrated Western tools. Even the cross- cultural validity of existing clinical interview methods are problematic [39]. A clinical gold standard is essential to get insight in the clinical relevance of scales. Understanding how clients view the change in the scales can be clarified through qualitative research or other informants in addition to the monitoring scales. Currently, the scale ratings give a general impression. They do not contain different modalities or different perspectives. Neither do they measure clinical risk factors, or control for response sets such as social desirability.

Traditionally, the efficacy of a treatment mode has been evaluated by statistical analyses involving comparisons of group means. However, group means represent averages that tell little about the variability of individual outcomes within a sample [40] [41] [42]. Among the alternatives suggested the method introduced by Jacobson and colleagues [42] is considered most useful [43]. According to this method a difference between pre- and post-measurement is clinically significant when a client at pre-test belonging to a disordered group has moved to the group (distribution) of normals by means of the intervention. Ideally, norms are available to define a cut-off score. When norms are absent, the researcher sets cut-off scores at either 2 Standard Deviations below the disordered average or 2 Standard Deviations above the normal mean. Next to this clinically significant criterion, it is argued that the amount of change should exceed the margin of measurement error. This can be determined by the Reliable Change Index [40].

The client’s rating at the beginning of the last session may influence the counsellor’s final assessment done at the end of the last session. In general we have managed this limitation appropriately by including a very large number of clients and counsellors in our analysis. In future research it may be desirable to blind the counsellor for the client’s final session rating.

The validity of the scales requires further research. The lack of a reference population masks the analysis of the scales’ ability to discriminate between client and non-client. Furthermore, it hinders assessment of the ability to compare change between client and control groups over time. To assess this other (comparative) objective measurements is also necessary. Intra and inter-rater reliability of the counsellors’ rating has been compared during training but not assessed formally, which limits the strength of the conclusions. Further research into the use of scales should address this through testing the intra- and inter- counsellor reliability.

## Conclusion

The analysis of a generic method to evaluate treatment in areas of conflict and disaster shows that it is possible to register in an easy way individual client changes without imposing Western, often non-validated, diagnostics and tools on the clients and counsellors. We find that rating scales measure significant changes over time. Analysis revealed that both client and counsellor rating scales had good criterion and concurrent validity when compared to each other. The rating scales showed that counsellors and clients perceive the usefulness of the treatment in rather identical positive ways.

Though these results need confirmation, we strongly suggest continuing to develop evaluation research that uses real-time, on-going client evaluation such as we described. The client, counsellor treatment feedback approach we describe is a promising instrument to improve treatment evaluation for regular psychosocial, mental health programmes in humanitarian settings.
